# The best marker for guiding the clinical management of patients with raised intracranial pressure—the RAP index or the mean pulse amplitude?

**DOI:** 10.1007/s00701-016-2932-z

**Published:** 2016-08-27

**Authors:** Allan Hall, Roddy O’Kane

**Affiliations:** 1 School of Medicine, University of Glasgow , University Avenue, G12 8QQ Glasgow, UK; 2 Department of Neurosurgery, Institute of Neurological Sciences, Queen Elizabeth University Hospital, G51 4TF Glasgow, UK

**Keywords:** Intracranial pressure, RAP index, Mean pulse amplitude

## Abstract

Raised intracranial pressure is a common problem in a variety of neurosurgical conditions including traumatic brain injury, hydrocephalus and intracranial haemorrhage. The clinical management of these patients is guided by a variety of haemodynamic, biochemical and clinical factors. However to date there is no single parameter that is used to guide clinical management of patients with raised intracranial pressure (ICP). However, the role of ICP indices, specifically the mean pulse amplitude (AMP) and RAP index [correlation coefficient (R) between AMP amplitude (A) and mean ICP pressure (P); index of compensatory reserve], as an indicator of true ICP has been investigated. Whilst the RAP index has been used both as a descriptor of neurological deterioration in TBI patients and as a way of characterising the compensatory reserve in hydrocephalus, more recent studies have highlighted the limitation of the RAP index due to the influence that baseline effect errors have on the mean ICP, which is used in the calculation of the RAP index. These studies have suggested that the ICP mean pulse amplitude may be a more accurate marker of true intracranial pressure due to the fact that it is uninfluenced by the mean ICP and, therefore, the AMP may be a more reliable marker than the RAP index for guiding the clinical management of patients with raised ICP. Although further investigation needs to be undertaken in order to fully assess the role of ICP indices in guiding the clinical management of patients with raised ICP, the studies undertaken to date provide an insight into the potential role of ICP indices to treat raised ICP proactively rather than reactively and therefore help prevent or minimise secondary brain injury.

## Introduction

The accurate measurement of dynamic intracranial pressure is complex. Although the measurement of ICP is an important factor in determining both the clinical management and outcome of patients with several neurosurgical conditions, recent studies have looked at the use of measuring the compensatory reserve as a possible predictor of clinical outcome and how it could be used to guide clinical management. Czosnyka et al. designed a coefficient between the mean intracranial pressure and the ICP mean pulse amplitude, the RAP index, as a potential descriptor of neurological deterioration in traumatic brain injury (TBI) patients [1]. Since this publication it has also been used to characterise the compensatory reserve in patients with hydrocephalus [2]. More recent studies have researched the use of the RAP index as a possible marker to guide clinical management in patients with raised ICP. This has included analysing the effects of baseline effect errors on both the RAP index as well as the ICP wave parameters, including the ICP mean pulse amplitude (AMP) [3, 4]. These studies concluded that baseline effect errors may hinder the use of the RAP index as a marker to guide clinical management due to their influential effect on the mean ICP, which is one of two factors used to calculate the RAP index. Other studies have shown that the AMP, as opposed to the RAP index, is refractory to these baseline errors and may provide a more accurate marker to guide clinical management, as well as its potential to predict mortality in certain neurosurgical events including traumatic brain injury [5].

This review will give an overview of intracranial pressure including its monitoring and examine the evidence from previous studies regarding the potential role of both the RAP index and the AMP as a marker to guide clinical management.

## Intracranial pressure

### Physiology

**Fig. 1:**
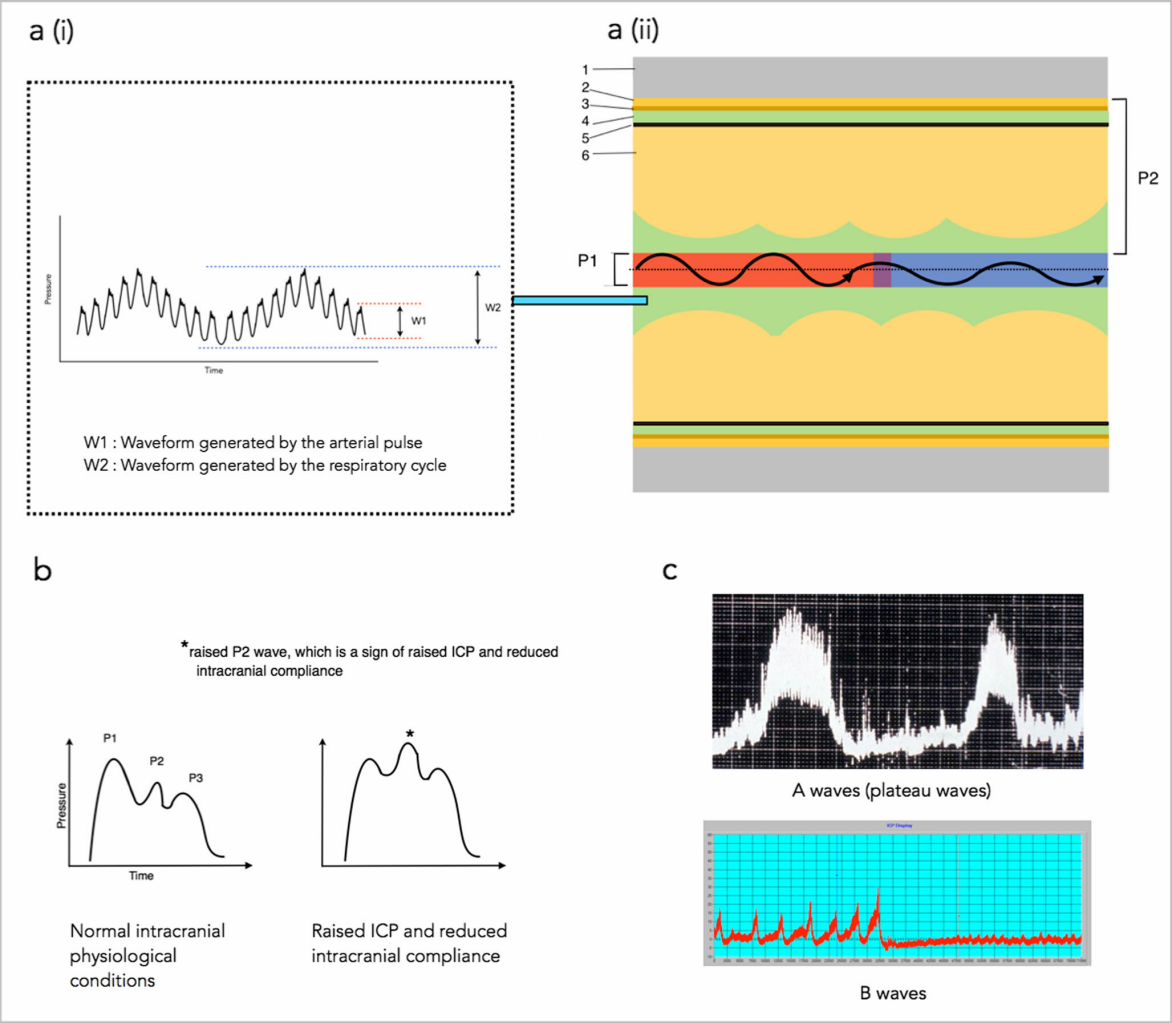
**An illustration of the ICP waveform components**. **a (i)** The ICP waveform showing the cardiac component (W1) and respiratory component (W2). The cardiac component consists of three peaks: P1 (reflects arterial pulsation), P2 (reflects, to a degree, intracranial compliance) and P3 (reflects aortic valve closure). The respiratory component reflects the cerebral venous pulsation generated by the changes in intra-thoracic pressure that is generated by respiration. **a (ii)** An illustration of what the individual ICP wave form peaks represent in terms of intracranial physiology. The black oscillating arrow represents arterial pulsations (red area), which subsequently become venous pulsations (blue area) as cerebral blood travels through the cerebral vasculature. P1 is the pressure generated in the CSF as a result of the pressure generated from the arterial pulsation. P2 is the pressure generated within the CSF as a result of the forces generated from both the pulsatile blood vessel and the resistance from the intracranial tissue. The higher the resistance is, the greater the pressure within the CSF and therefore the greater the P2 wave amplitude. Note the intracranial compliance, and therefore the value of P2, can be affected by any factor from the endothelium of the cerebral blood vessel to the intracranial surface of the skull (represented by P2) *Diagram key: 1, skull; 2, dura layer; 3, arachnoid layer; 4, subarachnoid space containing the cerebrospinal fluid (represented in green); 5, pia layer; 6, cerebral tissue*. **b** The individual ICP waveform peaks. Under the normal intracranial physiological state, P1 > P2 > P3. However when ICP is raised, and there is reduced intracranial compliance, P1 < P2 > P3. **c** The slow vasogenic waves of ICP, secondary to autoregulation-derived cyclic fluctuations of arterial blood volume. A waves, or plateau waves, always indicate pathology and signify reduced intracranial compliance. Once they are present, the patient will likely have neurological deterioration. They involve a steep rise in ICP from around a normal value to 50 mmHg or more. They persist for 5-20 min after which there is a sharp decline. B waves are more subtle than A waves and can indicate several abnormalities of intracranial homeostasis including being an indicator of failing intracranial compensation. It is important to note however that they can also be present in patients with normal ICP. B waves are rhythmic oscillations with ICP rising in a crescendo manner to levels 20–30 mmHg higher than baseline, with a subsequent sharp decline. They occur every 1–2 min. Note, Lundberg A waves and B waves refer to raises in static (mean ICP) and are not related to the pressure fluctuations during the cardiac cycle, which is the case for ICP wave amplitudes

Intracranial pressure (ICP) is derived from the circulatory dynamics of the CSF and cerebral blood flow that occur within the rigid intracranial compartment [6]. This relationship forms the ICP waveform, which consists of a cardiac component, which involves three peaks, a respiratory component, which is a reflection of the respiratory cycle on cerebral venous blood flow, and slow vasogenic waves of ICP, secondary to autoregulation-derived cyclic fluctuations of arterial blood volume (Fig. 1). ICP is affected by many factors both within and outside of the intracranial compartment.

Under normal circumstances ICP is kept within a certain range primarily with cerebral auto-regulation, which is itself dependent on cerebrovascular reactivity. The fact that true ICP is influenced by many factors and has both inter- and intra-patient variability makes accurate measurement of true ICP difficult. For example, the value of ICP is affected by age, body posture, time of day as well as the clinical condition of the patient. However a normal range of ICP has been established in order to address the multi-variant nature of ICP, as well as general interventional thresholds for ICP in different neurosurgical conditions.

**Fig. 2:**
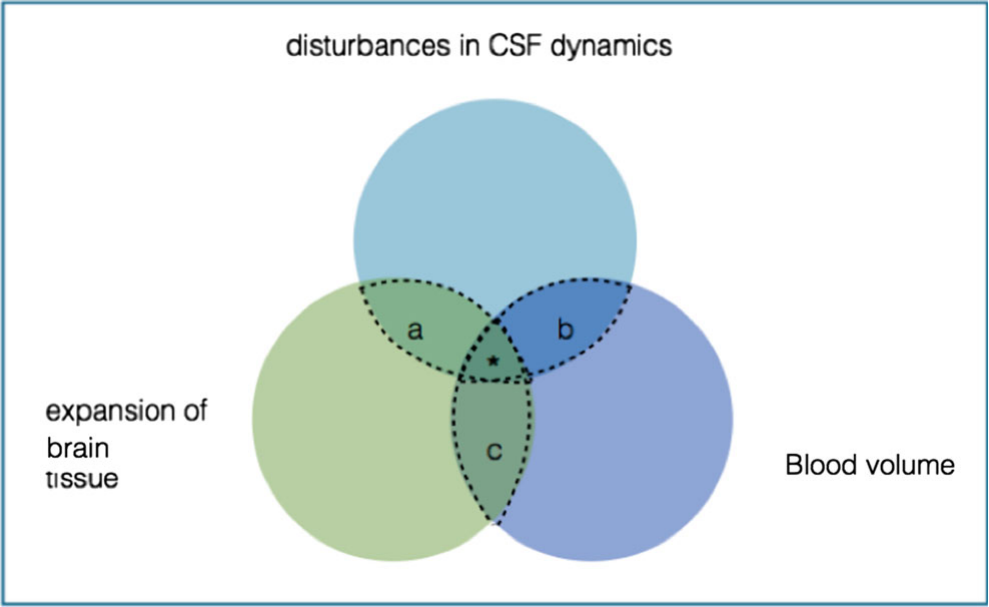
**Intracranial factors that affect ICP: the Monro-Kellie Doctrine**. Each circle represents one of the three broad intracranial categories: blood, CSF and brain tissue. If the normal physiological and/or anatomical state of either one of these categories is altered, and the compensatory change from the other categories is exhausted, this will result in an increase in ICP. The overlap of the circles represents how each category can affect the other (a, b, c). The density of the circles correlates with the ICP, with * denoting the highest ICP

Intracranial factors that can affect ICP can be broadly categorised into three main intracranial areas: disturbances in the dynamics of CSF; the expansion of intracranial tissue itself; the expansion of blood volume (Fig. 2). Disturbances within these intracranial areas can be caused by several factors including disturbances in the normal vascular architecture (resulting in either haemorrhage or mass effect), infection, malignancy (including metastasis) and trauma.

Extra-cranial factors are diverse and more difficult to manage from a neurosurgical point of view. For example, both the intra-thoracic compartment and, to a lesser extent, the intra-abdominal compartment influence ICP, in both the acute and chronic setting. In particular, the respiratory system plays a crucial role in the cerebral circulatory dynamics. This is evident by the role of ventilation to help reduce the vasodilatory effects of CO_2_ in patients with TBI [7, 8].

### Measurement and monitoring

Although understanding the homeostasis of ICP and its relationship to intracranial events is vital, the accurate measurement of true ICP is crucial in understanding its role in abnormal intracranial physiology and its effect on clinical outcome.

**Fig. 3:**
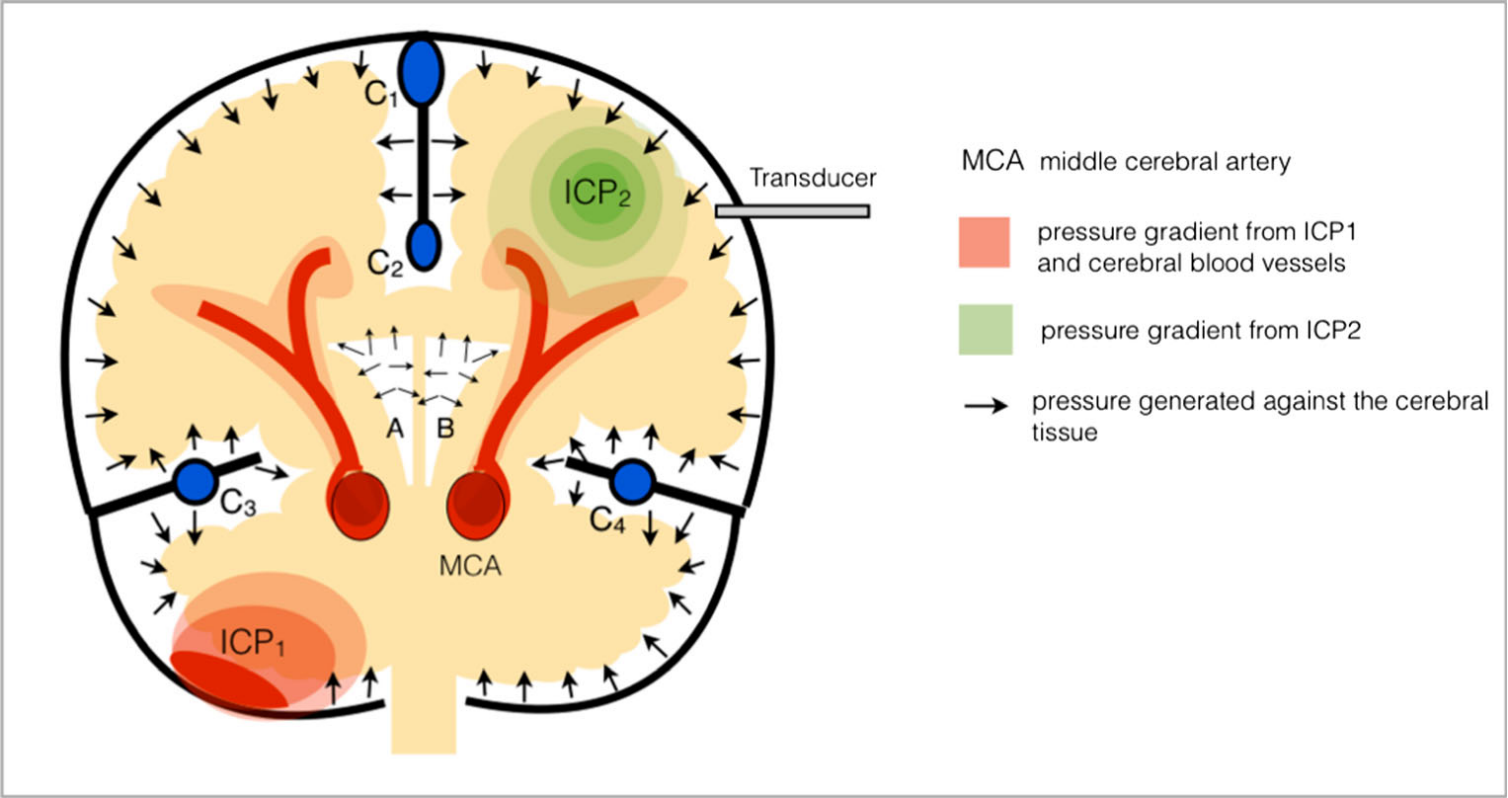
**The effect of pressure gradients on ICP monitoring.** If the transducer is placed in the frontal-parietal region, as shown, the effect of the pressure gradients generated by event 1 (ICP1), for example cerebellar haematoma within the infra-tentorium, has the potential to be different from the effect of the pressure gradient that is generated by event 2 (ICP2), for example TBI. The three-dimensional pressure gradients generated against the cerebral tissue itself is what will ultimately affect cellular function—the core issue in raised ICP. Note that the cerebral blood vessels also generate pressure gradients, which are a result of both cerebral blood flow and cerebral blood volume, and are influenced by cerebral vascular reactivity. *Diagram key: C1, superior sagittal sinus; C2, inferior sagittal sinus; C3 and C4, transverse sinus, A and B, lateral ventricles*

Measuring ICP is conventionally undertaken using either an intra-parenchymal or intra-ventricular transducer. It has been hypothesised that measuring intracranial pressure from a single source is itself a source of inaccuracy due to the complex three-dimensional pressure gradients that are established as a result of the complex intracranial architecture and physiology (Fig. 3). Studies have shown there is regional cerebral homeostasis as a result of regional effects on vasculature from local factors including metabolic and neural factors [9]. This local effect on regional vasculature is driven by cellular events ranging from increased production of waste products from cellular metabolism to changes in local neuronal activity. The reactivity of cerebral blood flow (CBF) to metabolic demand is not only rapid but is not always proportional [9]. This regional homeostatic effect has the potential to be another factor that could affect the true ICP values when measuring the ICP from a single source. However due to the invasive nature of ICP monitoring, accurately measuring pan-cranial ICP using the current invasive methods would be impractical.

Non-invasive methods of monitoring ICP have been assessed, including MRI and ultrasonography [10]. In particular the use of ultrasonography to measure optic nerve sheath diameter to assess dynamic changes in ICP has shown promising results [11]. Although no method has currently been found to be a suitable substitute for the more invasive method of ICP monitoring [12], further study into its use as a method of monitoring ICP from a variety of intracranial points would be useful, in particular, the potential role of using imaging techniques including fMRI to assess cerebral function and thereby establish the full relationship between raised ICP and changes in cerebral function. However the heterogeneity of cerebral vascular reactivity caused by ischaemia-induced impairment of cerebrovascular dynamics, including neurovascular coupling, could potentially hinder the use of fMRI in accurately assessing cerebral function in conditions that disrupt the blood-brain barrier such as in TBI.

## Intracranial pressure indices

### The RAP index

Czosnyka et al. designed an index that is a correlation coefficient between the mean ICP and the ICP mean wave amplitude (AMP)—the RAP index [correlation coefficient (R) between AMP amplitude (A) and mean pressure (P); index of compensatory reserve]. This was first introduced as a potential descriptor for neurological deterioration in TBI patients. It was subsequently used to characterise the pressure-volume compensatory reserve in patients with hydrocephalus. The RAP index has been shown to be a reliable measure of the compensatory reserve; however in more chronic states the cerebrospinal elasticity coefficient (E1) and AMP have been shown to express the compensatory reserve better [13–16]. This is due to the fact that raised ICP in chronic states, such as hydrocephalus and intracranial hypertension, is due to increased cerebral venous outflow or increased resistance to CSF outflow rather than the exhaustion of the cerebrospinal compensatory reserve. The RAP index has also been shown to be an accurate measure of intracranial compliance [17]. In addition, the RAP index has been used to assess the use of extra-ventricular devices to investigate the role of cerebral auto-regulation disturbances in aneurysmal subarachnoid patients [18].

**Fig. 4:**
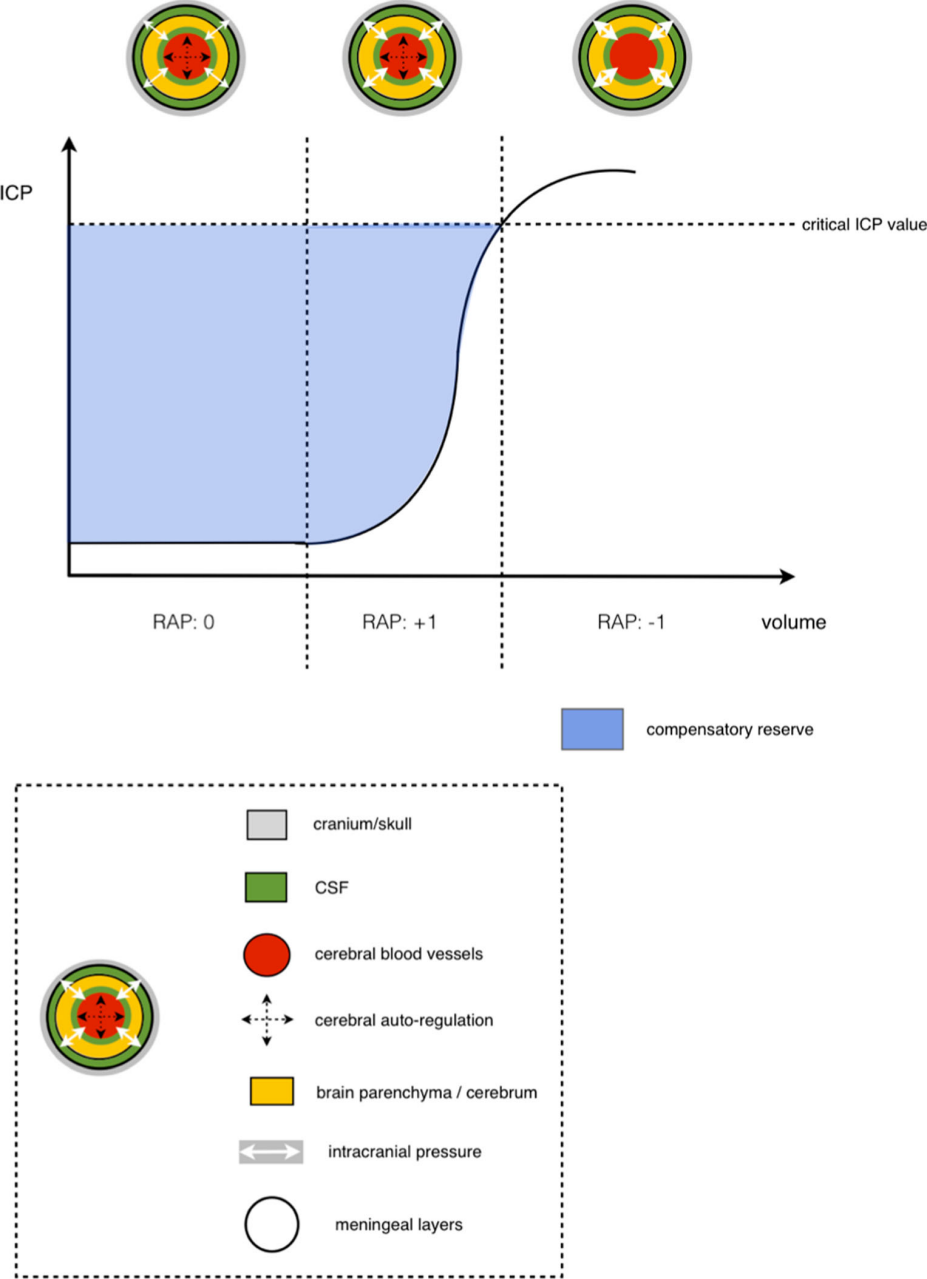
**The relationship among the pressure-volume curve, RAP index and cerebral auto-regulation.** When the RAP index is close to 0, cerebral auto-regulation remains intact and there is good compensatory reserve. As the ICP continues to rise and the RAP index approaches +1, the compensatory reserve decreases; however cerebral auto-regulation remains. When the ICP reaches the critical value, cerebral auto-regulation is lost and there is no compensatory reserve

The numerical value of the RAP index can range from +1 to -1 (Fig. 4). An RAP value of 0 is interpreted as lack of synchronisation between the changes in AMP and mean ICP. In terms of the pressure-volume relationship, an increase in intracranial volume does not result in a rise in ICP.

When the RAP index rises to +1, there is synchronisation between the rise in AMP and the rise in ICP. In terms of the pressure-volume relationship, a small rise in intracranial volume results in a larger rise in ICP.

If the ICP continues to rise, the RAP index value becomes -1. At this point the AMP has an inverse relationship to ICP—the AMP decreases as the ICP continues to rise. At this stage the compensatory reserve is exhausted and cerebral auto-regulation collapses. It is at this point that cerebral ischaemia- induced irreversible brain damage will likely occur and, ultimately, brain herniation. Note however that cerebral ischaemia will rise in a gradient fashion as the ICP continues to rise.

More recent studies have researched the potential role of the RAP index as a marker to guide clinical management [2, 19, 20]. However these studies found that the use of RAP as a potential marker to guide clinical management may be hindered by baseline effect errors. In particular it was found that baseline effect errors had a significant influence on the mean ICP as to render it an inaccurate reflection of the true ICP. This in turn influences the RAP index, which is calculated by correlating slow fluctuations of ICP and pulse amplitude.

### ICP mean pulse amplitude (AMP)

The ICP mean pulse amplitude relates to the pressure fluctuations of the ICP during the cardiac cycle. The AMP increases as the ICP rises; however once the critical ICP has been reached there will be subsequent derangement of cerebrovascular reactivity and AMP will decrease.

More studies are beginning to research the use of AMP both as a more accurate marker of the true ICP, in place of the mean ICP, as well as a tool to help guide the clinical management of patients [21, 22]. The influence that baseline effect errors have been shown to have on the mean ICP has raised questions as to how accurately the mean ICP value reflects the true underlying ICP. In particular it has been shown that AMP can make a useful contribution to the diagnosis of hydrocephalus as well as predict a positive outcome after shunt insertion [23]. However its use as a single parameter to guide clinical management has not yet been justified.

Although it has been demonstrated that AMP is refractory to baseline effect errors, further study into what other factors could potentially affect AMP, and therefore its usefulness in clinical management, must be investigated. This includes investigating the extent to which any factor influences AMP across the different pathological conditions. For example, studies have shown that AMP is more dependent on pulse changes in arterial blood volume in patients with TBI than in those with normal pressure hydrocephalus [24].

Further studies have assessed the AMP in relation to clinical outcome and have shown that a lower AMP is associated with a better outcome following TBI [25] and surgery [26].

One proposed advantage of using AMP instead of the RAP index is its ability to reflect the dynamic nature of true ICP without influences from static values, which have the potential to be affected by other dynamic factors such as baseline effect errors. However one limitation of using the AMP is the ability of researchers to accurately analyse the multi-variant and dynamic nature of the ICP waveform in an objective way. Studies have looked at more sophisticated algorithms of objectively analysing ICP wave parameters, including AMP, with a degree of success.

For example, it has been shown that the time domain method is superior to the frequency domain method for ICP waveform analysis since the frequency domain method can underestimate the AMP in the presence of heart rate variability or if the waveform has a high harmonic distortion [27]. Holm et al. [27] showed that the difference in the AMP calculated from these two methods was significant, with a difference of ≥2.0 mmHg in more than half of the 50,978 6-s time windows analysed. Furthermore, this difference will also affect the RAP index since the AMP is one of its components. Therefore, it is important that a standard method of analysis for the AMP is used in order to achieve robust results that can be accurately interpreted, which is important if both the RAP index and AMP are to be used to guide clinical management.

More recent studies have used more sophisticated algorithms to analyse the individual ICP waveform peaks (P1, P2, P3) with a high degree of accuracy.

However the successful clinical use of such algorithms for accurate, objective analysis of Lundberg waves, in particular B waves, has still to be proven [28, 29]. The proactive treatment of raised ICP instead of reactive treatment is one potential area where ICP waveform parameters, including AMP, could prove useful [30–32].

## Baseline effect errors

Baseline effect errors (BEEs) are spontaneous shifts in baseline pressure that occur during continuous ICP monitoring. They were first described by Eide in 2006 [33] when monitoring ICP simultaneously from two separate sensors in which there was a marked difference in mean ICP, combined with close to identical ICP waveform components such as AMP.

Baseline effect errors can be caused by a variety of factors ranging from all components of the ICP monitoring system, including the sensor, cable, display and transducer, to imperfect fluid connection caused by debris or air bubbles.

In relation to ICP indices, this is very important since any ICP index that includes mean ICP in its component will be affected, including the RAP index. However since the AMP is included in the RAP index, the effect of BEE is less pronounced in the RAP index than in the mean ICP. Therefore, any ICP index that is solely based on static pressure measurements can expect to be affected by BEE.

The ability of baseline effect errors to influence the value of the RAP index, and therefore the true nature of the patients underlying intracranial compensatory reserve, has the potential to affect clinical management. It has been shown that significant differences in the RAP index correspond to the occurence of BEEs and that the differences are of a magnitude that could affect patient management [3]. For example, an inaccurate RAP index could give the clinician a false reflection of the patient’s underlying intracranial physiological state and therefore instigate an erroneous change in their management that could range from altering doses of certain medications to undertaking inappropriate surgical intervention.

Baseline effect errors also have the potential to affect cerebral perfusion pressure, through the effect it has on intracranial pressure (since CPP is equal to mean arterial pressure minus intracranial pressure). For example, if BEEs are caused by an imperfect fluid connection as a result of debris or air bubbles, this could result in a fall in CPP if the ICP rises beyond the limits of cerebral auto-regulation.

## Current guidance in the clinical management of raised ICP

Raised ICP is defined as a sustained ICP of greater than 20 mmHg. However the thresholds for management depend, in part, on the underlying cause. At present there is no single parameter that has been proven to be the best marker to guide the clinical management of raised ICP. Due to the multi-variant nature of ICP between conditions and between patients it is doubtful whether it is possible to have one single parameter that can successfully guide the clinical management. The current treatment options for raised ICP range from initial medical management involving general physiological homeostasis to management, which involves more radical interventions including endotracheal intubation, barbiturate coma, and surgical evacuation or decompression. The ultimate goal of treating raised ICP is to prevent or minimise secondary brain injury by restoring intracranial homeostasis in order to reach optimum cerebral function.

### Treatment options

#### General physiological homeostasis

General physiological homeostasis involves controlling several parameters, which include blood pressure, serum osmolarity, temperature and blood glucose, as well as maintaining adequate partial arterial pressures of oxygen.

Blood pressure control has an important role in the treatment of raised ICP; however the exact treatment thresholds required to achieve optimum cerebral function in the setting of raised ICP remains ambiguous. This is in part due to the fact that patients with raised ICP often have several contributing factors. An important consideration in the control of a patient’s blood pressure within the context of raised ICP is the fine balance required between under-treatment of hypertension, and therefore increasing the possibility of haemorrhage or mass effect, and over-treatment of hypertension, and thus increasing the risk of ischaemia-induced damage to cerebral function. One therapy that aims to reduced the risk of ischaemia-induced cerebral damage is cerebral perfusion pressure (CPP)-guided therapy. This therapy aims to maintain CPP greater than 70 mmHg in order to minimise ischaemia, which is induced by compensatory cerebral vasodilation [34–36]. However CPP-guided therapy is not conclusive as it has been shown that this approach can cause a rise in ICP [37].

Another approach used to control blood pressure is the “Lund Protocol” in which a disruption of the blood-brain barrier is assumed. This places emphasis on reducing the hydrostatic forces whilst increasing the osmotic forces to maintain fluid within the intravascular compartment and thus prevent vasogenic oedema [38].

In addition to ensuring adequate CPP, oxygen levels must be adequately maintained, particularly in patients who have suffered TBI or intracerebral haemorrhage (ICH) in which the presence of pericapillary oedema may reduce the diffusion ability of oxygen to cross the blood-brain barrier.

Another important therapeutic target in raised ICP is glycaemic control. It has been shown in several studies that hyperglycaemia increases both the morbidity and mortality in patients with several neurological conditions including ICH and traumatic brain injury (TBI) [39–42, 43, 44]. Several of the underlying reasons for this increase in morbidity and mortality are the widespread detrimental effect that hyperglycaemia has on the cardiovascular system. However it has also been shown that hyperglycaemia causes a rise in ICP [45]. In particular, the breakdown of the blood-brain barrier that occurs in TBI patients results in the influx of glucose into the cerebral tissue thereby causing an increase in oedema through osmosis.

Minimising pyrexic episodes in patients with raised ICP is very important as an increase in temperature results in a rise in the cerebral metabolic rate, which can precipitate ischaemia directly, or indirectly through oedema. This is evident by studies that have shown, through experimental models of brain injury, the detrimental effect fever has on clinical outcome [46, 47]. Furthermore, the incidence of fever in patients who suffer basal ganglionic and lobar ICH, particularly ventricular haemorrhage, is high. Fever is also an independent predictor of poor clinical outcome after severe head injury. Therefore aggressive treatment of fever in patients with raised ICP is encouraged.

#### Head of bed elevation

Elevation of the head to 30° improves jugular venous outflow and reduces ICP. However caution must be used in patients who are hypovolemic as elevation in these patients could result in a fall in CPP, which could have significant effects on cerebral function, depending on the volume and duration of the drop in CPP.

#### Analgesia and sedation

Analgesia is important in order to minimise the potential harmful effect on ICP. Sedation may be used in order to appropriately ventilate patients who are unable to achieve adequate ventilation on their own. However this must be titrated with care in order to minimise the detrimental effect it may have on the clinician’s ability to clinically evaluate the patient.

#### Neuromuscular blockage

Patients who fail to respond to analgesia and sedation, and who still have intracranial hypertension, may benefit from neuromuscular blockade as seizure activity causes a rise in intra-thoracic pressure, which can obstruct cerebral venous outflow resulting in an increase in ICP. One of the drawbacks of this therapy is the masking of seizure activity.

#### Osmotic agents

One of the most important physiological properties that must be carefully balanced in patients with raised ICP is serum osmolarity. Both mannitol and hypertonic sodium chloride are the most common agents used.

Mannitol acts through two mechanisms. First, it increases intravascular volume and results in a reduction in blood viscosity. The intravascular volume expansion results in an increase in cardiac pre-load and thus an increase in CPP. Through cerebral auto-regulation, compensatory cerebral vasoconstriction occurs, which reduces the ICP. Second, mannitol draws water from the extracellular cerebral tissue into the plasma, thereby reducing cerebral oedema. However if the blood-brain barrier is disrupted, care must be taken to avoid worsening any cerebral oedema.

Hypertonic saline (HTS) can be used because of its osmotic effects as a result of its hypertonicity compared to extracellular cerebral tissue. However hypertonic saline also has haemodynamic, vaso-regulatory, immunological and neurochemical effects [48]. One recent study looked at the difference in cumulative and daily ICP burdens after severe traumatic brain injury between hypertonic saline and mannitol and found that HTS was more effective than mannitol when given as a bolus therapy. However the 2-week mortality rates between the two were not statistically different [49].

#### Steroids

The use of steroids in raised ICP is limited to treat vasogenic oedema where the underlying cause is primary and metastatic tumours. Steroids have shown no benefit in TBI or spontaneous intracerebral haemorrhage [50, 51].

In some studies they have been shown to have a detrimental effect [52, 53].

Steroids can also be used in CNS infection; however care should be taken in intracerebral abscesses as they can retard the encapsulation process, worsen necrosis and reduce the permeability of antibiotics.

#### Hyperventilation

The use of CO_2_ influence on cerebral auto-regulation provides one of the most effective methods for rapidly lowering ICP. This is achieved through ventilation, which allows the clinician to lower CO_2_ levels and induce compensatory cerebral vasoconstriction and thus reduce the ICP. One caveat to using this property of CO_2_ is the ability of the extracellular space of the brain to accommodate the associated changes in pH, and thus once CO_2_ levels begin to normalise a compensatory increase in ICP can occur. Therefore the main limitation of hyperventilation is its short therapeutic duration.

#### CSF drainage

CSF drainage is an effective method of lowering ICP in patients who have an intraventricular catheter to monitor ICP [31]. This is achieved by drainage of small amounts of CSF for short periods of time in response to elevations in the ICP. The main risks associated with ventriculostomy are infection and haemorrhage, with the incidence of bacterial meningitis varying between 6 % and 22 % [54, 55, 56].

#### Barbiturate coma

The use of barbiturates to induce a coma state is only suitable for refractory raised ICP. Barbiturates work by reducing the cerebral metabolic activity and thus reduce cerebral blood flow. However there are several potential side effects of this therapy including hypotension [57, 58]. Therefore careful monitoring of the patient’s haemodynamic state is vital.

#### Surgery

The role of decompressive craniotomy in the presence of a compressive pathology (such as subdural haematoma) is well documented. However its role in the absence of a compressive pathology is less well documented.

A number of studies, including the International Surgical Trial in Intracerebral Haemorrhage (STICH) trial, showed no real benefit in treating supra-tentorial ICH with craniotomy. Of those who were treated, a higher Glasgow Coma Scale (≥9) and a clot within 1 cm of the surface were associated with a 29 % relative benefit in functional outcome when compared with medical management [59–61]. Furthermore surgical intervention can provide more effective management in certain conditions such as diffuse, severe TBI and middle cerebral artery infarction than medical management [62].

However these studies did not include patients where the haemorrhage was located in the cerebellum. Further studies that did look at cerebellar ICH provided the conclusion that large cerebellar haemorrhages that either compress the brain stem or obstruct the fourth ventricle should be surgically removed as soon as possible [63, 64–69].

With regards to the timings of when surgery should be performed, there is no clear guidance on this [70]. The most supportive evidence is for operative removal within 12 h; however this is not conclusive.

Furthermore, bifrontal decompressive craniectomy in post-traumatic intracranial hypertension has been associated with significant reductions in AMP and has increased the compensatory reserve and therefore led to an improvement in the patient’s RAP index. [71] Although decompressive craniectomy has been reported to be beneficial in a number of conditions there is currently insufficient evidence to prove that it has the potential to improve outcome, despite showing benefits under certain conditions [72, 73].

## The role of the RAP index and AMP in future clinical management

ICP indices were introduced to help improve the diagnostic value of ICP. One of the main reasons for using ICP indices in patients with raised ICP is to allow clinicians to treat raised ICP proactively rather than reactively. The RAP index has already been used as a descriptor of neurological deterioration in TBI patients. However it has yet to be used to guide clinical management. One of the main reasons the RAP index may not be a suitable marker to guide clinical management is its susceptibility to baseline effect errors. Given the dynamic nature of ICP in conjunction with the varying causes responsible for baseline pressure effects, it is little surprise that the use of a static value of ICP (the mean ICP) may not provide the most accurate measure of true, global ICP. One appeal of using AMP instead of the RAP index as a marker for guiding clinical management in patients with raised ICP is the fact that the AMP has been shown to be uninfluenced by baseline effect errors. However one limitation of using AMP, and other parameters of the ICP waveform, as a marker to guide clinical management is the lack of appropriate tools to accurately analyse the ICP waveform objectively. However more sophisticated algorithms are making this less of a problem, such as the MOCAIP (morphological clustering and analysis of continuous intracranial pressure) method [19], which has shown high accuracy in analysing several ICP waveform parameters [55].

It is clear from the literature to date that the use of either the RAP index or the AMP as a marker to guide clinical management has its limitations.

However given the complexity of ICP, and the heterogeneity of the intracranial conditions that it is affected by, it is difficult to imagine that the RAP index or AMP alone will allow the clinician to make an informed decision that will optimise patient outcome, without other factors being involved including patient history, examination findings, basic neuro-imaging, haemodynamic and biochemical markers.

**Fig. 5:**
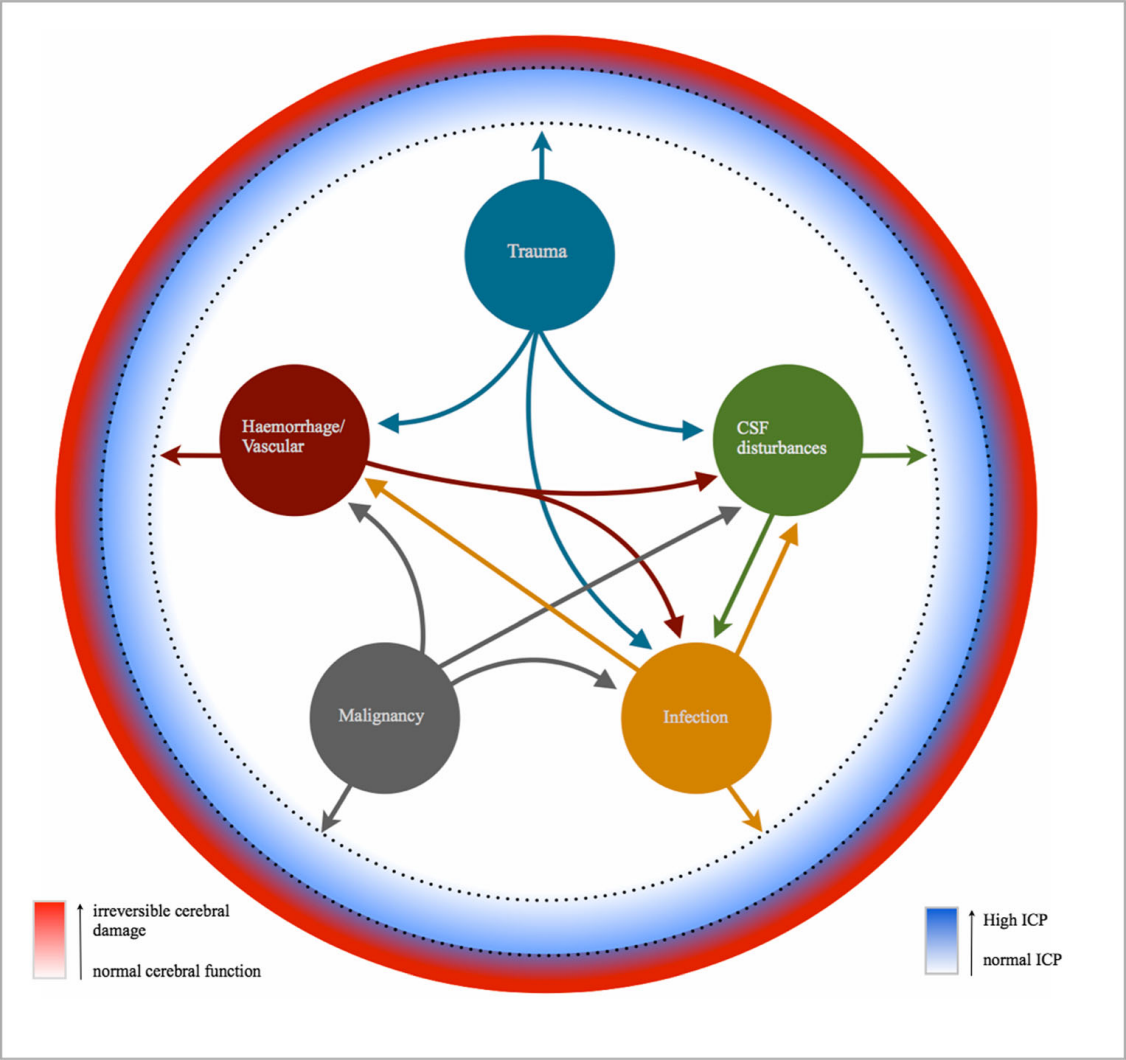
**The relationship between the different intracranial pathological categories and the ICP, cerebral function, RAP index and AMP.** Intracranial pathologies can be categorised into five main areas. The large circles represent the initial/primary pathology, with the arrows showing the potential secondary pathologies that can arise from the corresponding initial/primary pathology. For example, trauma can cause a direct increase in the ICP through the expansion of the cerebral parenchyma; however trauma can also result in damage to blood vessels with resulting haemorrhage, or a disturbance within CSF dynamics, e.g., expanding tissue resulting in blockage of the CSF drainage pathways or an increased risk of infection, which could cause a rise in the ICP through increased metabolic turnover. Note that the initial/primary pathology may itself not cause a significant rise in ICP; however the resulting secondary pathologies could be responsible for the rise in ICP, for example a malignant tumour itself may not cause a rise in ICP; however if the surrounding blood vessels rupture then it will be the resulting haemorrhage that will cause the rise in ICP. The nature of the primary pathology will determine what potential secondary pathologies could arise. The dotted circumferential lines represent the interval at which the RAP index and AMP would be most clinically useful. If changes in the AMP and/or RAP index are detected through ICP monitoring before the patient exhibits any signs/symptoms, then this could allow the clinician to implement a change in management sooner and therefore minimise irreversible damage to the brain tissue and subsequently improve patient outcome

The interval between the onset of raised ICP and just prior to the onset of cerebral damage is where ICP indices could prove most beneficial (Fig. 5).

## Conclusion

Raised ICP is in essence the result of cumulative molecular interactions, which establish three-dimensional multi-variant vectors within the intracranial cavity. This in turn generates pressure gradients that are exacerbated by the rigidity of the encompassing skull.

A sound understanding of this complexity of ICP and how it is affected by intracranial conditions is essential to achieve successful clinical outcome. In addition, as the intracranial cavity is essentially a highly dynamic and organised collection of cells and molecules, the use of mathematical and statistical modelling as a tool to accurately quantify and interpret intracranial dynamics is important.

The important role of minimising disturbances in ICP on a patient’s clinical outcome is evident and several studies have improved the ability to use certain ICP parameters to help achieve this. However it remains unclear which of these parameters, specifically, is best in predicting clinical outcome. Both the RAP index and AMP are currently used as markers for certain intracranial physiological parameters; however neither have yet been used as a marker to guide clinical management. Further studies into this potential role of the RAP index and AMP, including current barriers, are warranted.
